# Digging out with a robot

**DOI:** 10.1155/S1463924696000156

**Published:** 1996

**Authors:** Milton Levenberg

**Affiliations:** Structural Chemistry Department, D-418 Pharmaceutical Products Division Abbott Laboratories Abbott Park IL 60064-3500 USA


Journal of Automatic Chemistry, Vol. 18, No. 4 (July–August 1996), pp. 149–152

### Digging out with a robot

**Milton Levenberg**
*Structural Chemistry Department, D-418, Pharmaceutical Products Division, Abbott Laboratories, Abbott Park, IL 60064-3500, USA*

*Chemists at Abbott Laboratories are totally dependent on a rapid return of spectroscopic data on their intermediates and synthetic products to ensure that their chemical reactions are on track. Eight years ago, chemists at Abbott often had to wait three days to a week to receive this data, and had to start new reactions without it. That this was an unacceptable situation was clearly recognized by the Abbott chemists, and the management of the spectroscopy area. This paper discusses the approach used to provide totally automated sample preparation and running of both NMR and mass spectrometric samples. In both cases, systems have been developed by which the chemists log their own samples into a database, and a Zymark robotics system does the entire sample preparation and insertion into the spectrometer. Spectra are plotted directly in the client's lab, and the spectroscopy staff need only provide samples to the robot, fresh supplies (solvents, tubes, etc.) and routine maintenance, but no actual sample handling or spectrometer operation. The effects of this automation on throughput, sample turnaround time, and cost per spectrum are presented.*

### Introduction

Nuclear magnetic resonance (NMR) and mass spectrometry are the two techniques used by all organic chemists to confirm success of each step in their syntheses of new compounds.

In 1986, Abbott laboratories were running 20 000 'routine' NMR samples/year and 8600 routine (probe) mass spectra, with turnaround times of four days and seven days respectively. Much of the effort and careful analysis that should have been directed to less routine samples was falling by the wayside to crank out the routine samples as fast as possible. As spectrometers and people were added, the number of samples submitted increased proportionally. Clearly, the needs of the chemists were not being met, and as capacity was added the chemists perceived an improved turnaround time and submitted a larger fraction of their samples. The number of NMR spectra produced by the lab had climbed from 2000 in 1980 to 4500 in 1983 and 20 000 by 1986!

The chemists wanted their results within hours so they could make decisions on whether to proceed with the next synthetic step. Obviously their needs were not being fulfilled. A symptom of this was that many clients were asking for spectrometer time to run their own samples, with the expectation that by running their own samples they would ensure a quick return of their results.

Technology was ripe to attempt to automate sophisticated tasks. Computer programs had been written to process NMR data, an engineer was on the staff with expertise in barcode technology, and Zymate systems (Zymark Corporation, Hopkinton, MA) were doing simple, repetitive tasks. Most important, however, is that there were people on the staff with the skills and the desire to undertake this project, while still fulfilling other work commitments.

### Implementation

Once the scope of the project was defined, it was presented to management for approval and support. With the promise of more results with less incremental resources, it was not hard to obtain funding. At this point, Zymark became an active participant in fleshing out the details of the project plan. Many detailed discussions ensued between the staff and Zymark engineers to work out how their technology could be adapted to the task at hand. As a result of these discussions, and with a detailed definition of the requirements, it was decided that Zymark would do the robot design, primarily from their off-the-shelf modules, and they would write the initial version of the robot software.

Abbott personnel wrote the software to interface between the GE QE-300 NMR spectrometer, the database and log-in system, and the Zymate robot. Part of this project included programs to off-load the raw data from the NMR spectrometer to Abbott's VAX computer network for calculation and archiving. A design criterion necessary to improve throughput was that all calculations were done offline, and the spectrometer was dedicated to only acquiring the data. Figure 1 shows the calendar time required by each phase of this project. The numbers alongside the bars are estimates of the actual man hours devoted to each task. Figure 2 shows the layout of the first NMR robot system.

*Steps in processing a sample*

(1) The customer logs in the sample from *anywhere* (anywhere with a computer terminal and network connection or modem).

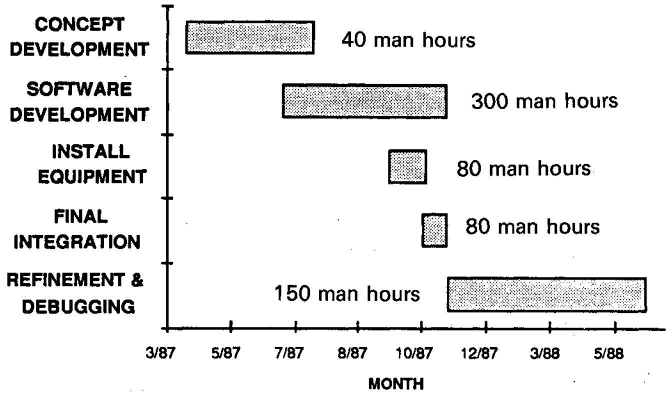

*Figure 1. Timeline for robot development.*

0142-0453/96 $12.00 © 1996 Taylor & Francis Ltd.

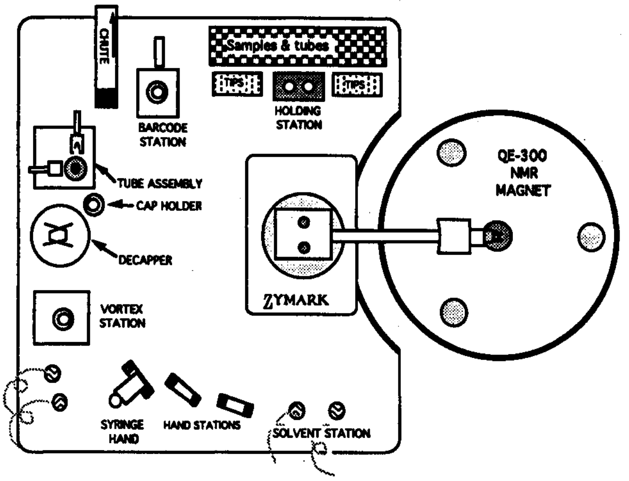

*Figure 2. Lay out of the first Zymark robot at Abbott Laboratories designed for preparing NMR samples.*

(2) The customer brings the sample to the NMR lab and adds a barcode label.
(3) The lab staff move the sample to the robot rack (and later retrieve it). This is the *only* interaction that the lab staff have with either the particular sample or its data. No other operator intervention is required, other than feeding supplies to the robot.
(4) The robot picks the samples in sequence from the rack, prepares the sample, and inserts the sample in the magnet when the spectrometer is free.
(5) The spectrometer shims the magnet and runs the sample.
(6) The raw data is off-loaded and archived.
(7) The computer network automatically processes the spectrum and plots it on a plotter in the client's lab.

*Sample preparation—the robot at work*

(1) A clean NMR tube from the sample rack is inserted into the NMR tube spinner in the tube assembly station.
(2) The robot picks up the next sample from the rack.
(3) The sample is placed in the barcode reader and identified.
(4) The sample is placed in the decapper, and the cap removed and placed in the cap holder station.
(5) The sample vial is placed under the appropriate solvent filling station and 0·7 ml of solvent metered out. The customer may choose between four solvents when the sample is logged into the system.
(6) The sample vial is placed on the vortexer to assist in dissolution.
(7) The vial is moved back to the decapper to hold it at a fixed location.
(8) The robot deposits the gripping hand in a hand station and picks up the pipetting hand.
(9) The robot and pipetting hand acquire a disposable pipette tip from the tip rack.
(10) The sample solution is pipetted to the clean NMR tube waiting in the tube assembly station. The disposable tip is ejected down the chute to the trash.
(11) The gripping hand is reacquired and the tube, with sample, is placed, in the holding station until the magnet is free.
(12) The cap is screwed back on the sample vial and the vial returned to its position in the sample rack.
(13) When the spectrometer signals the control computer that the data acquisition is completed, the old sample is ejected from the magnet and placed in the holding station.
(14) The freshly prepared sample is loaded into the spectrometer magnet.
(15) The old sample is moved to the tube assembly station where it is removed from the NMR tube spinner and placed in the sample rack.

Every attempt has been made to anticipate possible errors in the operation, and to provide programming to recognize when one of these errors occurs and provide a recovery path. Typical errors and recoveries include:

(1) No sample in rack—wait and try again in two minutes.
(2) Unable to read the barcode—discard sample and continue with the next one.
(3) No pipette tip in next rack position—search entire rack for one.
(4) Cannot assemble fresh NMR tube into spinner—reposition hand and try again.

A robot control program allows the operator to query the state of the robot control code and restart the sequence from various logical entry points.

Though not provided in the first automated system, the second generation system includes several routines created to provide capabilities to manage problems. A message reporting utility can be interrogated to read errors written by any of the routines responsible for handling samples or data. This allows the staff to quickly pinpoint the source of a problem when one occurs.

Routine upkeep and maintenance on the NMR system is as simple as keeping supplies of clean NMR tubes, solvents and pipette tips available to the robot. Four times a year the robot positions should be fine-tubed. The mass spectrometer requires changing DCI filaments, source filaments, and oil in the vacuum pumps, and cleaning of the ion source and quadrupoles. The intervals for this maintenance range from a month to once every two years, depending on the item.

### Data management

An Oracle-based tracking system (Oracle Corporation, Redwood City, CA) has been created which gathers and retains pertinent data about each sample. This system contains a list of all users (new users can enter themselves in the table) with their lab notebook number(s), the location of the closest plotter to their desk, and the project to be billed for our services.

As each sample is logged in, the system tracks its sample number, approximate weight (how many acquisitions are needed for good signal-to-noise), appropriate solvent, submission date, etc. This is all information *about* the sample, not the actual sample data.

As soon as the sample data is acquired by the spectrometer, it is assigned a sequential NMR number and automatically off-loaded to an archiving system.

The original system was based on magnetic tape archives, but it has been recently converted to optical media. This has the advantage of allowing immediate access to data many months old, but still on-line, by lab staff or client chemists. Of course, the optical media is expected to demonstrate much higher long-term reliability than magnetic tapes. All old data has been ported over to the new archiving system.

All of the calculations necessary to transform the NMR free induction decay (FID) data to a finished NMR spectrum are also performed off-line and automatically. Included in these calculations are baseline correction, Fourier transformation, phase adjustment, integration, and labelling each spectrum with its sample identification and acquisition parameters. The final spectrum is plotted on the plotter closest to the client's laboratory. Before this project began, several commercially available NMR processing software packages were evaluated and discarded. Members of the NMR laboratory wrote their own processing software, dubbed ABNMR, which has been in use ever since. At this point the lab staff and clients have a number of computer programs available, including several commercial packages as well as the programs developed in house.

### Present state of automation at Abbott

The sample load in the NMR and mass spectrometry labs has continued to grow since the first robotic system was put on-line in 1988. Figure 3 shows the total number of NMR spectra generated each year. The system described above has handled 60% of the total workload until very recently, when two new totally automated NMR systems were completed and brought on-line.

Shortly after the automated NMR system came on-line, a project was initiated to automate a mass spectrometer. The same technologies and strategies were applied as were used successfully in NMR. Again a Zymark robot was selected, and a Finnigan SSQ-70 mass spectrometer (Finnigan Corporation, San Jose, CA) was chosen to generate the mass spectra. Since the chemists were particularly interested in a confirmation of the molecular weights of their samples, it was decided to run in a desorption chemical ionization (DCI) mode, with the sample delivered on a direct insertion probe. With the help of internal staff, Abbott engineers and machinists, a probe shuttle mechanism was designed and constructed. One microlitre of a sample solution is deposited on the probe tip which is then transferred into the vacuum system.

This system came on-line in 1990, and now runs over half of the mass spectra. A second automated mass spectrometer is scheduled to be assembled within the next two years.

Every NMR spectrometer in structural chemistry is automated, at least to the point where a number of samples in NMR tubes can be loaded in a rack and the computer instructed to process each sample with its own, unique experiment during the course of the day, the night, or the weekend. This capability maximizes utilization of these very expensive spectrometers. Five NMR spectrometers are automated to this level with Zymark systems and two with systems purchased from the spectrometer vendors. As

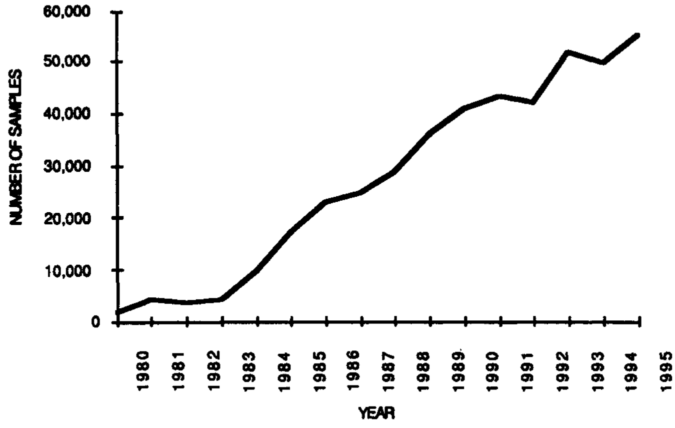

*Figure 3. Total number of samples run in Abbott's NMR lab each year.*

| | |
|---|---|
| Development | |
| Equipment cost | $290 000 |
| Robot upgrade | $70 000 |
| Manpower* | |
| 0·5 man years | $63 000 |
| Maintenance and operation total for eight years: | |
| Manpower* | |
| 0·2 * 8 man years | $293 000 |
| Supplies (solvents, tubes etc.) | $252 000 |
| *Total cost* | $968 300 |

The first automated NMR ran 190 600 samples in its first seven years.
The *cost per sample* is $968 300/190 600 = *$5 per sample.*

* Manpower cost as of the time of development of the system. It includes overhead, but not equipment depreciation.

*Figure 4. Cost analysis for Abbott's automated NMR system.*

| | |
|---|---|
| Development | |
| Equipment cost | $307 000 |
| Upgrades | $63 000 |
| Manpower* | |
| 0·5 man years | $67 000 |
| Maintenance and operation | |
| Manpower* | |
| 0·4 * 8 man years | $294 000 |
| Supplies (tips, filters, paper etc.) | $30 500 |
| *Total cost* | $761 500 |

The automated mass spectrometer ran 109 600 samples in five years.
The *cost per sample* is $761 500/109 600 = *$7 per sample.*

* Manpower cost as of the time of development of the system. It includes overhead, but not equipment depreciation.

*Figure 5. Cost analysis for Abbott's automated mass spectral system.*

mentioned above, three spectrometers are totally automated; and the newer systems have additional flexibility to allow the staff to prioritize samples and select a variety of experiments, including carbon 13 data acquisitions.

### Payback

A cost analysis of each of Abbott's first two systems is shown in figures 4 and 5.

### Summary and conclusion

Abbott's experience with automation of its more repetitive functions has been totally positive, and highly successful. With the current bill-out rate for lab time, these same samples that are averaging five to seven dollars to process on the robotic system would have cost 50 to 100 dollars each if run manually. The technology is certainly available to make a complex automation project a success; the key is to have a team of enthusiastic, motivated, capable people, and a co-operative and supportive vendor, to make the project succeed. Abbott will continue to automate in every appropriate situation.

